# Safety and pharmacokinetics of GSK3494245, a highly selective *Leishmaniasis* kinetoplastid proteasome inhibitor for the treatment of visceral leishmaniasis: A Phase 1, randomized, single ascending dose escalation study in healthy participants

**DOI:** 10.1371/journal.pntd.0014181

**Published:** 2026-05-13

**Authors:** Mischka Moodley, Michalis Kostapanos, Laura Iavarone, Marguerite Bracher, Silvia M. Lavezzi, Maria Davy, Pooja Singh, Ramya Bhat, Deborah Wong, Hema Sharma, Lionel K. Tan, Timothy J. Miles

**Affiliations:** 1 Global Health Medicines R&D, GSK, London, United Kingdom; 2 GSK Clinical Unit Cambridge, GSK, Cambridge, United Kingdom; 3 Department of Medicine, Addenbrooke’s Hospital, Cambridge University Hospitals and University of Cambridge, Cambridge, United Kingdom; 4 Clinical Pharmacology, Modeling and Simulation, Parexel, Milan, Italy; 5 Global Health, GSK, Stevenage, Hertfordshire, United Kingdom; 6 Clinical Pharmacology, Modeling and Simulation, Parexel, Dublin, Ireland; 7 Global Safety, GSK, Bangalore, Karnataka, India; 8 Biostatistics, GSK, Bangalore, Karnataka, India; 9 Global Clinical Delivery, R&D, GSK, Stevenage, Hertfordshire, United Kingdom; 10 Vaccines Clinical Sciences, GSK, London, United Kingdom; 11 Clinical Development, ViiV Healthcare Ltd., London, United Kingdom; 12 Global Health Medicines R&D, GSK, Tres Cantos, Madrid, Spain; CSIR-Indian Institute of Chemical Biology, INDIA

## Abstract

**Background:**

Visceral leishmaniasis (VL) is a neglected tropical disease caused by kinetoplastid parasites. If left untreated, VL has a case fatality >90%, making treatment essential for patient survival. Existing therapies have several limitations that impact treatment outcomes, including toxicity, requirement for parenteral administration, long treatment duration, and development of parasite resistance, driving the need for newer therapies. This Phase 1 first-time-in-human study evaluated the tolerability, safety, and pharmacokinetics (PK) of GSK3494245, a novel highly selective *Leishmania* kinetoplastid proteasome inhibitor.

**Methodology:**

This was a randomized, double-blind, placebo-controlled, single ascending dose study evaluating the oral administration of GSK3494245 in healthy participants aged 18 to ≤55 years (NCT04504435). The study was conducted at a single center in the United Kingdom between October 2020 and January 2024.

**Findings:**

GSK3494245 had an acceptable safety profile following administration of single doses of up to 240 mg in healthy male participants. All adverse events (AEs) were considered resolved or recovered, with most AEs being of mild or moderate severity. One serious AE of mild tachycardia was reported. The increase in exposure was slightly more than dose-proportional and approximately proportional under fasted and fed conditions, respectively. The median time to reach the maximum plasma concentration (C_max_) ranged from 0.50 to 1.79 hours, and the geometric mean of the elimination half-life ranged from 1.04 to 2.27 hours. Two participants met the C_max_ stopping criterion.

**Conclusion:**

GSK3494245 showed an acceptable safety profile over the dose range studied. However, based on its observed PK profile, GSK3494245 is unlikely to achieve the effective dose while ensuring safe exposure within the once- or twice-daily monotherapy target product profile recommended by the Drugs for Neglected Diseases initiative.

**Clinical Trial Registration:**

NCT Number (www.clinicaltrials.gov): NCT04504435

**EudraCT, CTIS**: 2019-004492-39

## Author summary

Visceral leishmaniasis (VL) is a serious parasitic disease that predominantly affects people in tropical and subtropical regions, including Brazil, India, and many African countries. It is transmitted via the bites of small insects called sandflies. The parasites attack important organs such as the spleen, liver, and bone marrow, leading to severe illness. Without treatment, VL is potentially fatal; however, with timely and appropriate therapeutic intervention, full clinical recovery is achievable. Current treatments are associated with poor tolerability, significant toxicity, and high costs, limiting their accessibility and feasibility in resource-limited settings. In this study, we tested a new oral treatment for VL (called GSK3494245) in humans for the first time. The treatment had an acceptable safety profile. Due to its properties, the drug would likely need to be taken three times a day to treat patients effectively and safely. An ideal treatment for VL would involve an oral medication that can be administered once daily and for a short duration, preferably less than 10 days. Therefore, this new oral treatment is currently unsuitable for patients with VL.

## Introduction

Visceral leishmaniasis (VL), also known as kala-azar, is a life-threatening parasitic disease caused by protozoan parasites of the genus *Leishmania* [1]. Transmitted through the bites of infected female *Phlebotomus* sandflies, VL affects organs such as the spleen, liver, and bone marrow. If left untreated, the mortality rate exceeds 90% [2,3]. The World Health Organization (WHO) recognizes VL as a neglected tropical disease, with an estimated 50 000–90 000 new cases of VL occurring annually worldwide [2]. More than 90% of cases are reported in Brazil, India, Ethiopia, Kenya, Somalia, South Sudan, and Sudan, although the disease remains endemic in more than 60 countries [4]. Despite declining incidence rates due to concerted public health efforts, VL remains a significant burden among some of the most vulnerable populations across the globe.

Currently, available therapies are limited and present several challenges, including prolonged treatment duration, parenteral administration, toxicity, teratogenic risks, and high costs [2,3,5–8]. Pentavalent antimonials, such as sodium stibogluconate, have been in use since the 1920s and require daily parenteral administration [9]. Their use is limited by severe toxicities, including pancreatitis, cardiotoxicity, and hepatotoxicity [4,10]. Liposomal amphotericin B (*AmBisome*), although effective, also requires parenteral administration, is costly, and can cause nephrotoxicity and infusion reactions, limiting its use to specific populations [7,10,11]. Paromomycin (*Humatin*) requires intramuscular injection, poses potential nephrotoxic and ototoxic risks, and its efficacy varies by region [12,13]. Oral miltefosine (*Impavido*) has limited effectiveness for VL, requires a 28-day regimen, and necessitates concomitant use of contraception due to teratogenicity concerns [10,14].

In response to the limitations of current treatments, as well as the emergence of drug resistance (particularly in South-East Asia) [9,13], research initiatives have driven the development of new pharmacological agents aimed at enhancing efficacy, reducing toxicity, and shortening treatment duration. LXE408 is a novel oral treatment currently being tested in Phase 2 clinical trials for patients with VL [15]. DNDI-6174, an inhibitor of *Leishmania* cytochrome bc1 complex, has also shown promise in animal models and has a favorable safety profile in the context of VL [16]. Candidates for cutaneous leishmaniasis include DNDI-6148 (oxaborole class) and DNDI-0690 (nitroimidazole class) [17–19].

Here, we describe a first-time-in-human (FTIH) study assessing GSK3494245, a novel, highly selective *Leishmania* kinetoplastid proteasome inhibitor and a potential candidate for short-course oral treatment of VL [20,21]. This Phase 1 study was designed to assess the safety, tolerability, and pharmacokinetics (PK) of GSK3494245 in healthy male participants, incorporating a food effect assessment.

## Methods

### Ethics statement

This study followed the ethical principles outlined in the Declaration of Helsinki. The study was approved by the Cambridge East Research Ethics Committee, Health Research Authority (Research Ethics Committee approval 20/EE/0184 granted on August 13, 2020). Written informed consent was obtained from all participants before enrollment. The study followed the guidelines of the International Council on Harmonisation (ICH) on Good Clinical Practice (GCP).

### Study design

This FTIH study was a randomized, double-blind (participant, investigator, and sponsor masking), placebo-controlled, crossover study of the oral administration of single ascending doses of GSK3494245 or placebo in healthy male participants under fasted and fed conditions (NCT04504435). The study was conducted at a single site in Cambridge, United Kingdom. The single ascending dose (SAD) part was initially planned to consist of two dose-escalation cohorts (cohorts 1 and 2) and a food effect cohort (cohort 3), each with a four-way crossover design, and an optional cohort with the same design (cohort 2A) to explore additional dose levels. In cohorts 1, 2, and 2A, each participant was to receive up to three single ascending doses of GSK3494245 or placebo under fasted conditions in a 3:1 ratio (8 participants per cohort). Cohort 3 investigated the effect of food, with participants receiving GSK3494245 or placebo under fasted vs. fed conditions in a four-way crossover design and in a 1:1:1:1 ratio (16 participants). The protocol was subsequently amended to include an additional cohort (cohort 3A) to assess single ascending doses of GSK3494245 under fed conditions, which followed the same four-way crossover design as cohorts 1 and 2 (8 participants). A maximum of eight dose levels was planned for the SAD part, commencing with a 20 mg dose.

The Dose Escalation Committee reviewed interim blinded safety, tolerability, and PK data after each dosing period to guide dose escalation decisions according to emerging data. Nonclinical studies conducted in various species, including mini pigs, supported the initiation of clinical studies in humans (with appropriate monitoring). Based on adverse histopathological findings in the liver and kidney of female mini pigs dosed at 300 mg/kg/day, the limiting exposure criterion was set at the no observed adverse effect level (NOAEL) of 120 mg/kg/day (60 mg/kg/dose), corresponding to a mean area under the curve (AUC) from 0 to 24 hours (0–24) of 48700 ng × h/mL and a mean maximum plasma concentration (C_max_) of 6100 ng/mL. A comprehensive list of stopping criteria and their rationale is provided in the Supporting Information (S1 Text). A multiple ascending dose (MAD) part was planned but not performed due to early study termination; hence, only the SAD part is described in this manuscript.

Eligibility criteria included participants being healthy males aged 18 to ≤55 years, with a body weight ≥50 kg and a body mass index (BMI) of 18.5–28 kg/m^2^, and no clinically significant medical conditions that could alter PK or safety or interfere with data interpretation. Since limited reproductive studies had been conducted with GSK3494245 at the time of the study, women of childbearing potential as well as pregnant women and nursing mothers were excluded from the study. A male participant with a female partner of reproductive potential had to agree to the use of contraception during the intervention period up to at least 90 days after the last dose of GSK3494245 and had to refrain from sperm donation for a similar period. A full list of inclusion and exclusion criteria is provided in the Supporting Information (S2 Text).

Screening occurred within 28 days before the first dose. On day 1, participants were assigned a unique randomization number in ascending numerical order. The randomization number encoded their assignment to GSK3494245 or placebo. All participants were randomized by GSK’s Statistics Department, using validated internal software (*Randall NG*) and following the Clinical Unit’s approved procedures. Each participant was administered the blinded study treatment under the supervision of the investigator or their designee at the site and following the planned dosing sequence.

Each cohort comprised up to four crossover intervention periods. Each intervention period consisted of a five-day (day -1 to day 4) inpatient stay at the Clinical Unit with a single dose of study intervention administered on day 1. Participants returned for a follow-up visit (which included a washout period) 7–14 days after the last study intervention administration.

Safety and tolerability were evaluated through adverse event (AE) reporting, physical examinations, vital signs, 12-lead electrocardiograms (ECGs), and continuous cardiac telemetry for 24 hours post-dose, as well as clinical laboratory findings.

The protocol and the statistical analysis plan of this FTIH study are available at: https://www.gsk-studyregister.com/en/trial-details/?id=208441#documents-section.

**Fig 1:**
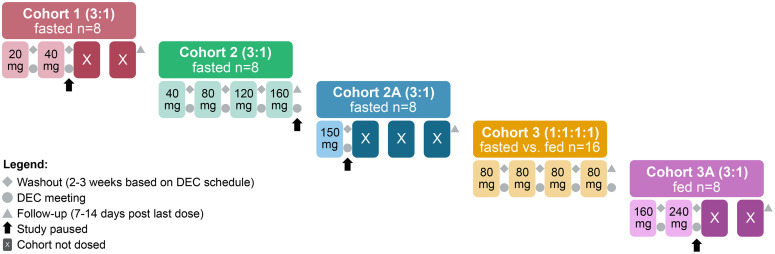
Cohorts dosed during the SAD study. **Abbreviations:** A = active drug (GSK3494245); C_max_ = Maximum Plasma Concentration; COVID-19 = Coronavirus Disease 2019; DEC = dose escalation committee; n = number of participants; P = placebo; SAD = single ascending dose. **Notes: In cohort 1 (red)**, participants received 20 mg (period 1) and 40 mg of GSK3494245 (period 2) or placebo under fasted conditions in a 3:1 ratio (6A:2P). Periods 3 and 4 did not take place due to a site closure associated with the COVID-19 lockdown. **In cohort 2 (green)**, participants received 40, 80, 120, and 160 mg of GSK3494245 (periods 1, 2, 3, and 4, respectively) or placebo under fasted conditions in a 3:1 ratio (6A:2P). **In cohort 2A (blue)**, participants received 150 mg of GSK3494245 or placebo (period 1) under fasted conditions in a 3:1 ratio (6A:2P). Periods 2, 3, and 4 did not take place due to a participant exceeding the C_max_ stopping criterion. **In cohort 3 (orange)**, participants received 80 mg of GSK3494245 under fasted conditions, 80 mg of GSK3494245 under fed conditions, placebo under fasted conditions or placebo under fed conditions in a 1:1:1:1 ratio (16A fed: 16A fasted:16P fed:16 P fasted). **In cohort 3A (purple)**, participants received 160 mg (period 1) and 240 mg of GSK3494245 (period 2) or placebo under fed conditions in a 3:1 ratio (6A:2P). A decision was made by the DEC not to proceed with further dose escalation following the 240 mg dose.

The following changes in study conduct were made in response to decisions by the Dose Escalation Committee (see Fig 1):

- Cohort 1: Dosing in periods 3 and 4 was canceled due to site closure associated with the coronavirus disease 2019 (COVID-19) lockdown. Therefore, participants in cohort 1 only received up to two dose levels of GSK3494245 (20 and 40 mg) under fasted conditions.
- Cohort 2: Participants received up to four dose levels of GSK3494245 (40, 80, 120, and 160 mg) under fasted conditions. One participant in cohort 2 met the C_max_ stopping criterion at 160 mg, and cohort 2A was therefore introduced following the same design with a dose level of 150 mg GSK3494245 under fasted conditions. Periods 2, 3, and 4 did not take place in cohort 2A due to another participant exceeding the C_max_ stopping criterion at the 150 mg dose.
- Cohort 3 (food effect cohort): On completion of cohort 3 (assessing GSK3494245 80 mg vs. placebo under both fasted and fed conditions in a 1:1:1:1 ratio), the decrease in C_max_ under fed conditions led to the introduction of cohort 3A to test additional dose levels of GSK3494245 vs. placebo in a 3:1 ratio taken with food. Only two dose levels (160 and 240 mg) were explored in this cohort. A dose of 160 mg was selected as the first dose in cohort 3A based on the following: (1) dosing with food in cohort 3 had demonstrated a lower C_max_ and a similar AUC compared with the fasted state for the 80 mg dose, (2) the exposure at the 160 mg dose was predicted to remain within the PK stopping criteria, and (3) there had been no safety signals in cohort 3.

The study was discontinued on 15 November 2023 due to the observed PK data not aligning with the requirements of the target product profile (TPP) for VL, as defined by the Drugs for Neglected Diseases initiative (DNDi) [22]. The clinical program was subsequently terminated following the final participant visit on 14 January 2024.

### Outcomes

The primary objective was to assess the safety and tolerability of GSK3494245 in healthy participants through evaluation of AEs as well as clinically significant changes from baseline in laboratory tests, physical examinations, vital signs, and cardiac monitoring (12-lead ECGs/telemetry). Serious adverse events (SAEs) and AEs were reported from the signing of the informed consent form and the administration of the first dose, respectively, until the follow-up visit. AEs and SAEs were followed from day 1 until the event had resolved, stabilized, was otherwise explained, or the participant was lost to follow-up.

The first two participants in each cohort acted as sentinels; following review of their first 24-hour post-dose safety data (e.g., vital signs, ECGs and AEs) by the principal investigator, the remaining participants were randomized.

The secondary objective was to assess the PK of GSK3494245 in healthy participants following a single oral dose under fasted and fed conditions. The following PK endpoints were calculated: the AUC from 0 to 24 hours (AUC[0–24]), to infinity (AUC[0-∞]), and to the time of last non-zero concentration (AUC[0-t]); the C_max_; the time to reach C_max_ (T_max_); and the elimination half-life (t½). Dose-proportionality (based on AUC[0-∞] and C_max_) and food effect (based on AUC[0–24], AUC[0-∞], and C_max_) were also assessed. Plasma samples were collected pre-dose and at 10 and 30 minutes, as well as at 1, 1.5, 2, 2.5, 3, 4, 5, 6, 8, 10, 12, and 14 hours (for cohorts 3 and 3A only), and at 24 hours after dosing on day 1.

For the full list of objectives, please refer to clinicaltrials.gov (NCT04504435).

### Sample size

The study was anticipated to include 40 participants in the SAD part, with eight participants each in cohorts 1, 2, and 3A, and 16 participants in Cohort 3. Additional participants were recruited to replace participants who were withdrawn. For cohorts 1, 2, 2A, and 3A, with a minimum of six participants per dose and a between-participant coefficient of variation (CV) of 30% for clearance, the 95% confidence intervals (CI) of the mean clearance per dose were estimated within 36% of the point estimate. For cohort 3, the sample size was based on the precision estimates for fed vs. fasted conditions, assuming a within-participant CV of 20% for AUC and 30% for C_max_.

### Pharmacokinetics & statistical analysis

PK parameters were calculated by standard non-compartmental analysis using *Phoenix WinNonlin* version 8.3 (Certara USA, Inc., Princeton, New Jersey, USA). All calculations of non-compartmental parameters were based on actual sampling times. The area under the concentration-time curve was calculated using the lin up/log down trapezoidal rule.

No formal hypothesis was tested. An estimand approach was used where appropriate, with point estimates and CIs provided. Analysis sets included screened, enrolled, safety, and PK sets. The safety set was defined as any randomized participants who received at least one dose of the study intervention, while the PK set was defined as any participants in the safety set with at least one non-missing PK assessment. All PK assessments were part of the secondary objectives based on the PK set. PK parameters were derived from plasma concentration-time data using standard non-compartmental analysis. Plasma concentrations and PK parameters were summarized descriptively. Graphical presentations were provided as appropriate. Statistical analyses were performed to assess the dose-proportionality (all cohorts) and food effect (cohort 3).

## Results

### Demographics and disposition

This study was conducted from 26 October 2020 to 14 January 2024 (when the last participant completed their final visit). The study was terminated following the completion of the SAD part. The study duration was extended due to recruitment delays attributed to the COVID-19 lockdown, the implementation of additional cohorts, and protocol amendments in response to emerging data.

**Fig 2:**
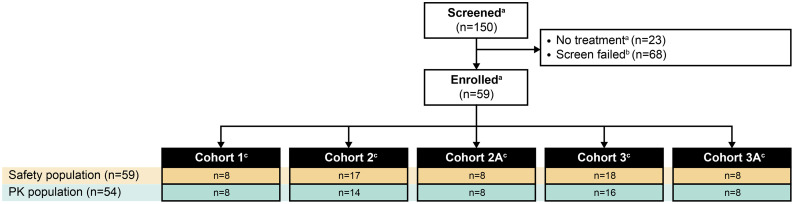
Participant flow. **Abbreviations:** n = number of participants; P = placebo; PK = pharmacokinetic. **Notes:** ^**a**^ The “screened population” included all participants who were screened for eligibility (n = 150). Participants who received “no treatment” (n = 23) met the eligibility criteria but did not enter the study. The “enrolled population” included all participants who passed screening and entered the study – this included the “randomized population” (n = 59). ^**b**^ Reasons for screening failure: (i) did not meet inclusion/exclusion criteria (n = 58); (ii) withdrawal by participant (n = 6); (iii) physician decision (n = 4). ^**c**^ **In cohort 1**, the dosing schedules in periods 1-2 were (i) 20 mg-P in Period 2; (ii) P-40 mg; (iii) 20-40 mg; and (iv) 20-40 mg, respectively. **In cohort 2**, the dosing schedules in periods 1-4 were: (i) 40-80-120 mg-P; (ii) 40-80 mg-P-160 mg; (iii) 40 mg-P-120-160 mg; and (iv) P-80-120-160 mg, respectively. **In cohort 2A**, the dosing schedules in period 1 were 150 mg; 150 mg; 150 mg; P**. In cohort 3**, the dosing schedules in periods 1-4 were: (i) P(fed)-P(fasted)-80 mg (fasted)-80 mg (fed); (ii) P(fasted)-80 mg (fed)-P(fed)-80 mg(fasted); (iii) 80 mg(fed)-80 mg (fasted)-P(fasted)-P(fed); and (iv) 80 mg (fasted)-P(fed)-80 mg(fed)-P(fasted), respectively. **In Cohort 3A**, the dosing schedules in periods 1-2 were: (i) P-240 mg; (ii) 160 mg-P; (iii) 160-240mg; and (iv) 160-240 mg, respectively.

A total of 59 participants (39% of the screened set) were enrolled in the SAD part of the study. Among these, 38 (64%) were withdrawn from the study. The most common reason for withdrawal was the physician’s decision (n = 26), followed by withdrawal due to an AE (n = 9). Withdrawals due to the physician’s decision included, among others, a dose escalation decision, i.e., the decision to withdraw participants due to the study being terminated at the end of cohort 3A (n = 8), the inability to proceed with dose escalation due to PK stopping criteria (n = 8), and a request from the Sponsor hepatic safety panel to initiate a new cohort with different participants (n = 7). The participant flow is presented in Fig 2.

All enrolled participants received at least one dose of the study intervention (GSK3494245 or placebo) and were included in the safety analysis set. In total, 54 (91.5%) participants had at least one PK assessment available and were included in the PK set. All participants were male, with a median age (minimum, maximum) of 36 years (20, 55) and a median BMI (minimum, maximum) of 25 kg/m^2^ (19.7, 29.1). The majority of participants were White (76%). No discernible differences in demographics or other baseline characteristics were observed across cohorts or study interventions (Table 1).

**Table 1:** Summary of participants’ demographic characteristics.

Parameters		SAD study (n = 59)
Sex (n [%])	Male	59 (100%)
Age (years) ^a^	Mean (SD)	36.1 (8.29)
	Median (min, max)	36.0 (20, 55)
Race (n [%])	White/Caucasian/European	45 (76%)
	De-identified ^b^	14 (24%)
Weight (kg)	Mean (SD)	78.36 (8.82)
	Median (min, max)	78.30 (61.0, 98.7)
Height (cm)	Mean (SD)	177.9 (7.84)
	Median (min, max)	178.0 (158, 193)
BMI (kg/m²)	Mean (SD)	24.74 (2.07)
	Median (min, max)	24.98 (19.69, 29.07)

**Abbreviations:** BMI = body mass index; Max = maximum; Min = minimum; n = number of participants; SAD = single ascending dose; SD = standard deviation

**Notes:** ^a^Age was imputed when the full date of birth was not provided; ^b^ Due to the small number of participants (n < 11), racial categories were combined to minimize the risk of participant re-identification.

### Safety

A summary of AEs ranked by overall frequency and system organ class (SOC) is presented in Table 2. Forty-one participants experienced at least one AE, and 90 AEs were reported in total. A single AE was considered related to the study intervention by the investigator; this was reported for one participant in cohort 1 (Period 1) who received placebo under fasted conditions. This AE was an increase in alanine aminotransferase (ALT) of moderate severity.

**Table 2:** Summary of AEs ranked by overall frequency and system organ class.

AE	Placebo	GSK3494245										
All cohorts (N = 48)	Cohort 1		Cohort 2				Cohort 2A	Cohort 3		Cohort 3A	
	20 mg fasted (N = 6)	40 mg fasted (N=6)	40 mg fasted (N = 6)	80 mg fasted (N = 6)	120 mg fasted (N = 6)	160 mg fasted (N = 6)	150 mg fasted (N = 6)	80 mg fasted (N=15)	80 mg fed (N = 15)	160 mg fed (N = 6)	240 mg fed (N = 6)
**Any AE**	27 (56%)	2 (33%)	3 (50%)	2 (33%)	1 (17%)	1 (17%)	2 (33%)	2 (33%)	5 (33%)	7 (47%)	5 (83%)	3 (50%)
**Treatment-related AE**	1 (2%)	0	0	0	0	0	0	0	0	0	0	0
**Withdrawal due to an AE**	6 (13%)	0	0	1 (17%)	0	1 (17%)	0	0	0	1 (7%)	0	0
**SAE**	1 (2%)	0	0	0	0	0	0	0	0	0	0	0
**Treatment-related SAE**	0	0	0	0	0	0	0	0	0	0	0	0
**Fatal SAE**	0	0	0	0	0	0	0	0	0	0	0	0
**AE by SOC**												
Cardiac disorders	2 (4%)	0	0	0	0	0	1 (17%)	0	0	0	0	0
Gastrointestinal disorders	6 (13%)	0	0	0	0	0	1 (17%)	1 (17%)	0	0	0	0
General disorders and administration site conditions	3 (6%)	0	1 (17%)	0	0	0	0	0	2 (13%)	0	1 (17%)	0
Hepatobiliary disorders	1 (2%)	0	0	0	0	0	0	0	0	1 (7%)	0	1 (17%)
Immune system disorders	1 (2%)	0	0	0	0	0	0	0	0	0	0	0
Infections and infestations	4 (8%)	0	0	0	0	0	0	0	2 (13%)	1 (7%)	1 (17%)	2 (33%)
Injury, poisoning, and procedural complications	1 (2%)	0	0	0	0	0	0	0	1 (7%)	1 (7%)	1 (17%)	0
Investigations	3 (6%)	0	1 (17%)	1 (17%)	0	0	0	0	0	1 (7%)	0	0
Metabolism and nutrition disorders	1 (2%)	0	0	0	1 (17%)	0	0	0	0	0	0	0
Musculoskeletal and connective tissue disorders	3 (6%)	0	0	0	0	0	0	0	0	2 (13%)	1 (17%)	0
Nervous system disorders	4 (8%)	2 (33%)	0	0	0	0	0	0	0	1 (7%)	2 (33%)	0
Renal and urinary disorders	1 (2%)	0	0	0	0	1 (17%)	0	0	0	0	0	0
Respiratory, thoracic, and mediastinal disorders	3 (6%)	0	0	0	0	0	0	1 (17%)	0	1 (7%)	0	0
Skin and subcutaneous tissue disorders	3 (6%)	1 (17%)	2 (33%)	1 (17%)	1 (17%)	0	1 (17%)	0	0	1 (7%)	1 (17%)	1 (17%)

**Abbreviations:** AE = adverse event; N = number of participants; SAE = serious adverse event; SOC = system organ class.

**Notes:** These data reflect the number and percentages of participants receiving a treatment reporting at least one AE and not the number of AEs reported for each treatment. Hence, the totals indicated within a header SOC may add up to less than the number of events reported within the column. Additionally, participants are categorized by treatment received at the time of experiencing an AE. Therefore, a participant could be counted across multiple treatments.

Most AEs were of mild or moderate severity. Two AEs involving increased blood creatine phosphokinase were reported as severe in two participants from cohort 3, including one participant who received 80 mg of GSK3494245 under fed conditions and one who received placebo under fasted conditions. The investigator identified strenuous physical activity as the underlying cause of the elevated serum creatine phosphokinase levels observed in these two participants; hence, these two severe AEs were considered unrelated to the study intervention. All AEs were considered resolved or recovered at the last follow-up visit of the study.

Nine participants were withdrawn from the study due to AEs. One SAE of mild broad complex tachycardia (grade 1), deemed unrelated to the study intervention by the investigator, occurred in a 42-year-old male participant who received placebo under fasted conditions (cohort 3). The participant, whose treatment sequence was GSK3494245 80 mg fed/fasted followed by placebo fasted/fed, reported this SAE on period 3, day 63 (i.e., 62 days after the first dose and 25 days after the last dose of GSK3494245). The participant remained stable and returned to a normal sinus rhythm without medical intervention. ECGs and telemetry were normal at screening and throughout the study until the above-mentioned event. There were no known underlying medical conditions that could have predisposed the participant to tachycardia, nor did the participant take any concomitant medication that could account for the event. Two participants met the dose adjustment/PK stopping criteria, specifically the C_max_ stopping criterion (see S1 Text), when receiving GSK3494245 160 mg (cohort 2) and 150 mg (cohort 2A), respectively. Neither of the participants who exceeded the C_max_ stopping criterion reported AEs at the time of elevated C_max_. One participant with an AE of non-sustained ventricular tachycardia met the individual participant safety stopping criterion. No fatal AEs were reported during the study.

Regarding safety laboratory chemistry assessments, some participants exhibited fluctuations in serum lactate, bicarbonate, and pH, typically following meals. A few participants experienced changes from baseline of chemistry-related laboratory parameters that were reported as AEs; however, none (with the exception of the increased ALT in a participant dosed with placebo) were considered related to study drug by the principal investigator. No other clinically concerning trends were detected in any hematology parameters or urine analysis. Three participants had clinically significant post-baseline changes in telemetry. The first participant in cohort 2 experienced a non-serious event of asymptomatic 7-beat broad complex tachycardia (non-sustained supraventricular tachycardia) recorded on telemetry approximately 24 hours post-dose (GSK3494245 160 mg). The participant was asleep at the time of the event and later reported vivid dreams occurring around the same time. No abnormal ECG changes were recorded in this participant during the current or previous dosing periods, nor during the extended 24-hour period following the event. The event was deemed unrelated to the study drug by the investigator. The second participant in cohort 2 (placebo) experienced an AE of ventricular extrasystoles. This participant had an asymptomatic high burden of premature ventricular contraction, with approximately 300 beats over a 6-hour period and brief episodes of ventricular trigeminy. The event occurred approximately 14 hours after dosing with placebo, while the participant was asleep. Telemetry was extended for an additional 24-hour period and did not reveal any similar findings. The third participant experienced an SAE of tachycardia, with details discussed previously.

### Pharmacokinetics

**Fig 3:**
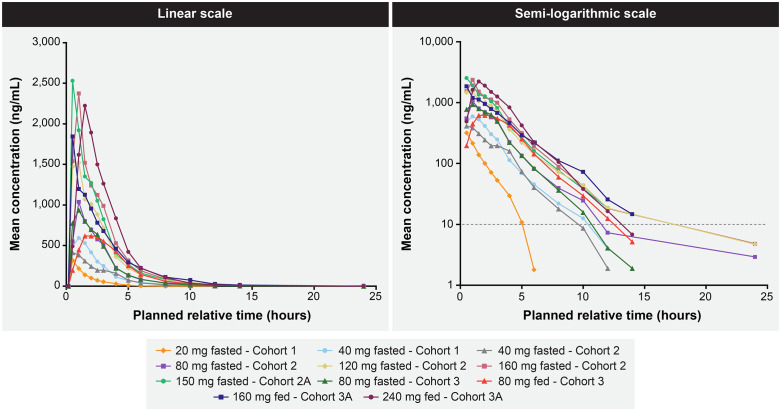
Mean plasma concentration-time profile of GSK3494245 after administration of single ascending doses. **Notes:** The mean plasma concentration (ng/mL) of GSK3494245 after administration of single ascending doses under fasted or fed conditions is shown relative to the planned time (hours) on linear (left) or semi-logarithmic (right) scales. The horizontal line on the semi-logarithmic scale (right) shows the lower limit of quantification of 10 ng/mL.

The PK profile of GSK3494245 following single-dose administration under fasted or fed conditions is presented in Fig 3; summaries of PK parameters are reported in Table 3. Notably, one participant receiving 150 mg of GSK3494245 under fasted conditions in cohort 2A had no quantifiable PK concentrations on both plasma and urine testing (with unknown etiology despite extensive investigation) and was therefore excluded from summary calculations.

**Table 3:** Summary of GSK3494245 PK parameters after single-dose administration for each cohort.

Cohort	Dose	Period	N	n	Parameter	AUC(0-∞) (h × ng/mL)	AUC (0-t) (h × ng/mL)	AUC (0–24) (h × ng/mL)	CL/F (mL/h)	C_max_(ng/mL)	T_max_^b^(h)	t½ (h)	Vz/F (mL)
Cohort 1	20 mg fasted	Period 1	6	6	GM	465.33	437.70	465.34	42980.10	303.10	0.50	1.04	64618.68
					% CV^a^	34.14	37.37	34.16	34.14	37.1	0.50, 0.53	7.32	29.57
	40 mg fasted	Period 2	6	6	GM	1398.60	1365.97	1398.13	28600.13	709.22	1.00	1.25	51616.98
					% CV^a^	63.53	63.71	63.51	63.53	31.8	0.50, 2.00	46.31	29.19
Cohort 2	40 mg fasted	Period 1	6	6	GM	1203.31	1164.38	1202.78	33241.72	525.73	0.75	1.71	82152.22
					% CV^a^	29.36	30.81	29.34	29.36	56.0	0.50, 1.50	30.20	29.37
	80 mg fasted	Period 2	6	6	GM	2491.31	2423.55	2488.16	32111.61	981.04	1.00	2.27	105285.78
					% CV^a^	58.09	59.39	58.21	58.09	75.7	0.50, 2.50	16.80	67.18
	120 mg fasted	Period 3	6	6	GM	4106.78	4034.57	4105.32	29220.00	1645.64	0.75	2.01	84885.57
					% CV^a^	50.70	51.08	50.70	50.70	83.3	0.50, 2.50	20.60	42.52
	160 mg fasted	Period 4	6	6	GM	5175.57	5103.90	5173.94	30914.50	2229.53	1.00	1.67	74437.57
					% CV^a^	58.92	59.96	58.96	58.92	101.9	0.50, 4.00	19.73	68.32
Cohort 2A	150 mg fasted	Period 1	6	5	GM	6129.38	6054.45	6127.36	24472.28	3033.94	1.00	1.84	64925.27
					% CV^a^	49.83	49.92	49.82	49.83	61.7	0.50, 2.00	8.98	44.67
Cohort 3	80 mg fasted	Period 1	4	4	GM	3487.41	3443.43	3485.38	22939.65	1785.33	0.75	1.91	63133.81
					% CV^a^	26.38	26.38	26.35	26.38	25.1	0.50, 1.50	17.02	18.76
		Period 2	4	4	GM	2188.16	2156.31	2187.76	36560.40	1174.29	1.25	1.72	90886.94
					% CV^a^	20.01	20.43	20.03	20.01	29.4	0.50, 2.50	30.47	44.72
		Period 3	4	4	GM	2649.24	2606.66	2649.17	30197.39	1063.68	0.75	1.67	72651.03
					% CV^a^	35.76	36.49	35.76	35.76	51.0	0.50, 1.03	11.59	28.57
		Period 4	3	3	GM	2557.47	2529.29	2557.44	31280.89	1059.32	1.00	1.32	59523.61
					% CV^a^	24.57	25.05	24.57	24.57	34.4	0.50, 2.00	4.72	26.60
	80 mg fed	Period 1	4	4	GM	2583.97	2554.45	2583.24	30960.11	950.87	1.75	1.64	73218.52
					% CV^a^	20.59	20.61	20.57	20.59	35.4	0.50, 2.00	26.22	9.16
		Period 2	4	4	GM	2231.84	2190.46	2231.16	35844.94	748.00	1.75	1.44	74263.29
					% CV^a^	46.16	46.77	46.12	46.16	22.5	1.00, 2.50	39.61	13.66
		Period 3	4	4	GM	3255.04	3206.24	3252.29	24577.27	850.78	1.79	2.05	72623.24
					% CV^a^	18.69	18.52	18.65	18.69	14.0	1.00, 2.50	14.60	12.51
		Period 4	3	3	GM	2456.76	2420.93	2456.66	32563.23	888.61	1.50	1.44	67826.64
					% CV^a^	47.32	47.62	47.34	47.32	44.0	1.00, 4.00	16.69	28.96
Cohort 3A	160 mg fed	Period 1	6	6	GM	4481.08	4428.67	4479.07	35705.67	2044.18	0.77	1.71	88216.43
					% CV^a^	39.91	39.23	39.87	39.91	58.7	0.50, 2.00	30.07	35.05
	240 mg fed	Period 2	6	6	GM	6549.60	6512.32	6548.63	36643.47	2488.65	1.50	1.50	79316.40
					% CV^a^	16.72	16.94	16.71	16.72	22.2	1.00, 2.00	17.13	6.45

**Abbreviations:** AUC(0-∞)=area under the curve (time 0 to infinity); AUC(0-t)=area under the curve (0 to time of last non-zero concentration); AUC(0–24)=area under the curve (0–24 hours); CL/F = apparent clearance; C_max_; = maximum plasma concentration; CV = coefficient of variation; GM = geometric mean; t½ = half-life; N = number of participants per treatment and period; n = number of participants with PK parameter; PK = pharmacokinetics; T_max_ = time to maximum concentration; Vz/F = apparent volume of distribution.

**Notes:** ^a^Between-participant coefficient of variation; ^b^ Values for T_max_ are median instead of geometric mean, and minimum, maximum instead of %CV.

GSK3494245 was rapidly absorbed into systemic circulation across all dose levels (Fig 3). A single concentration peak was observed at each GSK3494245 dose. Peak levels (C_max_) progressively increased at higher doses and were generally lower under fed vs. fasted conditions (Fig 3 and Table 3). Median T_max_ ranged from 0.50 to 1.79 hours, generally with a later peak under fed conditions (except for 160 mg GSK3494245 under fed conditions) (Table 3).

The geometric mean AUC(0–24) was similar to AUC(0-t) and AUC(0-∞) in each cohort, indicating that most absorption and distribution occurred within the first 24 hours post-dose, with only a negligible percentage of the AUC(0-∞) derived from extrapolation to infinity.

In most participants, GSK3494245 was cleared from systemic circulation by 10–12 hours post-dosing. The geometric mean t½ across dose levels was relatively short, between 1.04 and 2.27 hours. Geometric mean clearance was consistent across all dose levels and under fasted vs. fed conditions. The volume of distribution ranged from 51.62 to 105.29 L.

Under fasted conditions, C_max_ variability ranged from low to high, with %CV of 25.1% to 101.9%, and AUC(0-∞) variability was low to moderate, with %CV of 20.01% to 63.53%. Under fed conditions, PK variability was low to moderate overall, with C_max_ %CV of 14.0% to 58.7% and AUC(0-∞) %CV of 16.72 to 47.32%.

Two participants under fasted conditions exceeded the C_max_ stopping criterion threshold (6100 ng/mL), with one participant receiving 160 mg (C_max_ = 6280.4 ng/mL) and one participant receiving 150 mg (C_max_ = 8135.5 ng/mL) of GSK3494245. No participants had an AUC(0-∞) higher than the AUC stopping criterion threshold (48 700 h × ng/mL).

Dose-proportionality assessment showed that the increase in exposure under fasted conditions was slightly higher than dose-proportional, with slope estimates of 1.20 (90% CIs: 1.02, 1.38) for AUC(0-∞) and 1.15 (90% CIs: 0.93, 1.37) for C_max_ (Table 4). Under fed conditions, the increase in exposure was approximately dose-proportional, with slope estimates of 0.97 (90% CIs: 0.67, 1.27) for AUC(0-∞) and 1.10 (90% CIs: 0.80, 1.39) for C_max_.

**Table 4:** Analysis of the dose-proportionality of GSK3494245.

Condition	Parameter (units)	Slope log parameter vs. log dose	90% CI of the slope
Fasted	AUC (0-∞) (h × ng/mL)	1.20	(1.02, 1.38)
	Cmax (ng/mL)	1.15	(0.93, 1.37)
Fed	AUC (0-∞) (h × ng/mL)	0.97	(0.67, 1.27)
	Cmax (ng/mL)	1.10	(0.80, 1.39)

**Abbreviations:** AUC(0-∞)=area under the curve (0 hours to infinity); CI = confidence interval; C_max_ = maximum plasma concentration; PK = pharmacokinetics.

**Notes:** A power model was used to assess the dose-proportionality. Log (dose) and period were considered to be fixed effects, and the participant was considered to be a random effect. Unstructured covariance structures were used to account for both intra- and inter-individual variability. A slope of 1 indicates that PK increased linearly with the dose. A slope >1 indicates that PK increased more than proportionally with the dose.

The assessment of the food effect showed that the C_max_ of an 80 mg dose decreased by approximately 32% under fed compared with fasted conditions, with a geometric least squares mean (GLSM) ratio of 0.68 (90% CI: 0.59, 0.79) (Table 5). However, the GLSM ratios were 0.94 (90% CI: 0.85, 1.04) for both AUC(0-∞) and AUC(0–24). These data indicate that GSK3494245 administration under fed conditions significantly lowered C_max_ without impacting overall exposure.

**Table 5:** Analysis of the food effect on the PK values of GSK3494245 (cohort 3).

Parameter (unit)	Fed		Fasted		GSK3494245 80 mg fed vs. fasted			
n	LS mean	n	LS mean	Difference	90% CI	GLSM ratio	90% CI
AUC (0-∞) (h × ng/mL)	15	7.85	15	7.92	-0.06	(-0.16, 0.04)	0.94	(0.85, 1.04)
AUC (0–24) (h × ng/mL)	15	7.85	15	7.92	-0.06	(-0.16, 0.04)	0.94	(0.85, 1.04)
C_max_ (ng/mL)	15	6.74	15	7.12	-0.38	(-0.52, -0.24)	0.68	(0.59, 0.79)

**Abbreviations:** AUC(0-∞)=area under the curve (0 hours to infinity); AUC(0–24)=area under the curve (0–24 hours); CI = confidence interval; C_max_ = maximum plasma concentration; GLSM = geometric least squares mean; LS mean = least squared mean; n = number of participants per treatment; PK = pharmacokinetics.

**Notes:** The log-transformed parameter was modeled based on fed or fasted conditions, with the period considered to be a fixed effect and the participant considered to be a random effect. Unstructured covariance structures were used to account for both intra- and inter-individual variability. A ratio of 1 indicates equivalent PK exposure under fed and fasted conditions. A ratio >1 indicates a higher PK exposure under fed conditions. A ratio <1 indicates a lower PK exposure under fed conditions.

## Discussion

VL is a life-threatening disease for which current therapeutic options are both limited and suboptimal in addressing the disease burden [1]. DNDi has recently published a TPP for “ideal” or “acceptable” treatments for VL to ensure their accessibility and adaptability to resource-limited settings [22]. Characteristics of the ideal TPP include efficacy against resistant strains, suitability for all populations (including pregnant women and immunocompromised patients), and a once-daily oral administration with a maximum treatment duration of 10 days or via intramuscular injections administered three times over 10 days. A once-daily oral regimen supports adherence in outpatient care, while intramuscular injections provide a practical alternative for severe cases or when oral administration is unfeasible, ensuring effective treatment across diverse healthcare contexts.

GSK3494245 emerged as a potential candidate for VL treatment owing to its highly selective inhibition of the *Leishmania* proteasome [13,20,21]. Although the proteasome is conserved across kinetoplastid protozoa, including *Trypanosoma* species, GSK3494245 demonstrated superior *in vivo* efficacy in VL models [21,23]. By targeting the kinetoplastid-specific chymotrypsin-like activity—distinct from the mammalian proteasome—it delivers potent antiparasitic effects while minimizing off-target toxicity, offering a promising strategy for safer, more effective VL therapy. Ongoing studies are investigating the efficacy and safety of this mechanism of action in related compounds, such as LXE408 [21,23,24].

This Phase 1 FTIH study evaluated the safety, tolerability, and PK of single ascending doses of GSK3494245 in healthy participants under fasted or fed conditions. To enhance the robustness and validity of the study outcomes, the study design incorporated comprehensive safety monitoring and a dose escalation procedure as well as the use of double-blinding.

The findings of this study indicate that GSK3494245 exhibited an acceptable safety profile and was well-tolerated following the administration of single doses up to 240 mg in healthy male participants. All AEs reported in this study were resolved or recovered at the last study follow-up visit, with most being mild in severity and considered by the investigator to be unrelated to GSK3494245. Other than meal-associated fluctuations in serum lactate, bicarbonate, and pH, there were no trends of clinical concern amongst the safety laboratory parameters. A few participants experienced brief ECG/telemetry changes that resolved spontaneously; none of these were considered related to the study intervention.

GSK3494245 was rapidly absorbed into the systemic circulation, with C_max_, AUC(0-t), and AUC(0-∞) increasing in a slightly greater than dose-proportional manner under fasted conditions and in an approximately dose-proportional manner under fed conditions. GSK3494245 administration under fed conditions led to slightly delayed T_max_ and decreased C_max_, while the overall exposure (AUC) remained similar between fed and fasted conditions. This pattern is consistent with a food-induced delay in gastric emptying [25], which slowed the rate of drug delivery to absorption sites without significantly affecting the extent of absorption.

Given the dose-limiting C_max_, considerable PK variability under fasted conditions, and a short half-life, GSK3494245 is unlikely to meet efficacy and safety requirements within the recommended TPP as monotherapy administered once or twice daily. With the aim of developing a treatment suitable for primary healthcare settings and in alignment with field experience, administering three times daily dosing with meals is challenging to implement. Furthermore, reformulating GSK3494245 poses a challenge, with no guarantee that the desired PK and safety characteristics can be achieved. Our study highlights some of the challenges in developing new drugs that meet the ideal TPP for VL. High efficacy is crucial for improving outcomes, limiting the development of drug resistance, and addressing disease severity. Furthermore, an acceptable safety profile is critical for the overall tolerability of treatment regimens. The treatment should be suitable for combination therapy, a strategy increasingly used to enhance effectiveness in real-world settings and reduce the development of drug resistance [13]. These clinical requirements must be balanced with the need for a drug that is accessible and affordable in endemic regions [3]. Ensuring that treatments can be easily administered in low-resource healthcare settings is essential; however, modifying formulations to meet these requirements can introduce additional complexity into the drug development process [22]. GSK3494245 met certain criteria of the ideal TPP, including a favorable safety profile and suitability for oral dosing; however, its PK characteristics and the resulting need for two to three daily oral doses administered with food compromised its progression. Although not explicitly required by the DNDi TPP, administration under fasted conditions is better suited to VL treatment contexts where food availability is often limited and unpredictable.

Limitations of this study include its conduct in a homogeneous population of healthy male participants at a single site in the United Kingdom, which limits the generalizability of the safety and PK findings to the broader population, including patients with VL and women. Another limitation is that the study was terminated early, and thus the MAD part was not conducted. The short follow-up duration and limited sample size precluded assessment of long-term effects and chronic toxicity of GSK3494245. Dropouts due to COVID-19 lockdowns, along with the fact that meeting PK stopping criteria did not allow, as per protocol, four dose escalations within the same cohort (for cohorts 1, 2A, and 3A), may have limited the ability to evaluate intra-individual variability of PK parameters. Although the study provided information on single-dose safety, tolerability, and PK, no comprehensive data supporting the definition of an optimal dosing regimen of GSK3494245 was obtained.

Despite the limitations, this FTIH study offers key lessons for early-phase development in neglected tropical diseases. Early PK assessment against a stringent TPP is essential for enabling timely go/no-go decisions and preventing advancement of compounds unlikely to meet therapeutic requirements. Proactive termination based on clear evidence reflects ethical and scientific best practice, minimizing unnecessary participant exposure and conserving resources for more promising candidates. Furthermore, publishing outcomes from discontinued studies promotes transparency and informs future strategies—an especially critical consideration in resource-limited fields. Finally, this trial underscores that Phase 1 studies serve not only to assess safety but also as a pivotal checkpoint for feasibility, reinforcing their role in de-risking development and accelerating progress toward effective therapies.

In summary, this Phase 1 FTIH study provided insights into the safety, tolerability, and PK of GSK3494245, a novel highly selective kinetoplastid proteasome inhibitor that targets the *Leishmania* protozoan parasite. Despite apparently acceptable safety data, the study was terminated earlier than planned due to unfavorable PK characteristics that did not fully align with the DNDi TPP [22]. This evidence-based outcome supported an early decision to discontinue development of GSK3494245, thereby avoiding unnecessary progression to Phase 2 and beyond. The study highlighted some of the challenges faced by drug developers in meeting the established TPP criteria for new drugs targeting VL.

## Supporting information

S1 Text Stopping criteria for study treatment. (DOCX)

S2 Text Inclusion and exclusion criteria. (DOCX)

S1 Checklist CONSORT Checklist. Checklist of items in accordance with the CONSORT guidelines (adapted from https://dx.doi.org/10.1136/bmj-2024-081123).
(DOCX)
