# Supplementary material for: Safety and pharmacokinetics of GSK3494245, a highly selective Leishmaniasis kinetoplastid proteasome inhibitor for the treatment of visceral leishmaniasis: A Phase 1, randomized, single ascending dose escalation study in healthy participants
Source: PLoS Negl Trop Dis. 2026 May 13;20(5):e0014181. doi: 10.1371/journal.pntd.0014181 (PMC13245864; doi:10.1371/journal.pntd.0014181)
Supplement: S1 Text — (DOCX) [file pntd.0014181.s001.docx]

## S1 Text. Stopping criteria for study treatment

Discontinuation of study treatment was considered permanent. Discontinued participants were not allowed to re-enter the study. Study treatment restart or rechallenge, after stopping criteria had been met by any participant, was not allowed. All criteria for discontinuing, stopping, and escalating doses in this study complied with the most recent European Medicines Agency (EMA) guidelines (EMEA/CHMP/SWP/28367/07) available at the time of protocol development.

**Nonclinical findings supporting various clinical stopping criteria**

The systemic toxicity of GSK3494245 was evaluated in oral repeat-dose toxicity studies of up to 14 days duration in rats and mini pigs. All pivotal safety pharmacology and toxicology studies were conducted in an Organisation for Economic Co-operation and Development (OECD) member country in accordance with the OECD Test Guidelines and the principles of Good Laboratory Practice (GLP). Based on the histopathological findings, various stopping criteria were implemented for participant safety as described below.

Renal stopping criteria were implemented based on findings of renal tubular dilatation, tubular basophilia, and interstitial inflammatory cell infiltration in mini pigs. Liver chemistry stopping criteria were introduced due to inflammatory cell infiltration and hypertrophy of the portal and bile duct epithelium in rats, findings that were also observed in mini pigs. Olfactory stopping criteria were incorporated into the study following the observation of olfactory epithelial degeneration in the nasal turbinates of female rats dosed at 300 mg/kg/day. Mitochondrial toxicity stopping criteria were also implemented as an extra precaution, although isolated inhibition of adenosine triphosphatase (ATPase) synthetase was considered unlikely at the expected exposure for GSK3494245 in healthy human participants. Indeed, GSK3494245 demonstrated weak inhibition of isolated bovine ATP synthase, which accounted for the reductions in mitochondrial respiration observed in fresh, isolated rat liver mitochondria (up to 26% at 200 µM), with comparable effects anticipated in HepG2 cells.

### Dose adjustment and pharmacokinetics (PK) stopping criteria

The following dose adjustment and PK stopping criteria were applied:

- Dose escalation was stopped if any single participant reached exposures greater than PK stopping criteria exposure thresholds of the area under the curve from zero to infinity (AUC[0-∞]) of 48 700 ng×h/mL or maximum plasma concentration (C_max_) of 6100 ng/mL in the Single Ascending Dose (SAD) part of the study. Such thresholds were based on the no observed adverse effect level (NOAEL) for renal findings in female mini pigs. The PK dose escalation rule was used for progression to the next dose or dose adjustment. This involved establishing a relationship between exposures (AUC and C_max_) and dose levels using a power and linear mixed-effects model when PK data were available from at least two prior dose levels. This model was continually updated with new dose levels as they became available. This model was used to project exposure for the next dose level.
- If the 95^th^ percentile of the predicted exposure from the maximum total daily dose reached the PK stopping criterion exposure, dose escalation was stopped or dose adjustment was planned, as appropriate. Based on the emerging safety, tolerability, and PK information, the Dose Escalation Committee decided whether to evaluate any lower doses or to repeat the doses already evaluated in the remaining periods to collect additional data.

### Dose escalation and study stopping criteria

The principal investigator (PI) and the Sponsor Medical Monitor reviewed the following, and the study was stopped if any of these criteria were met:

- Two or more participants from the previous dose level on active treatment experienced an adverse event (AE) of severe intensity and reasonably attributable (in the PI’s opinion) to the dosing with GSK3494245.
- One or more participants in the study developed an adverse renal event, as defined in the renal withdrawal criteria (see below), considered to be related to GSK3494245 (in the PI’s opinion in consultation with the Sponsor Medical Monitor).
- One or more participants from any dose level experienced a serious adverse event (SAE) with a reasonable possibility of relation to the study product.

All other stopping criteria were applied even if no PK stopping criteria were met.

### Renal stopping criteria

A participant who met any of the following criteria was withdrawn from the study:

- New onset of any clinically significant and persistent (within 48 hours) hematuria, as confirmed by microscopy.
- New onset of clinically significant and persistent (within 48 hours) proteinuria (spot urine albumin creatinine ratio ≥30 mg/mmol) in the absence of another clinical explanation, e.g., calculus/infection.
- If (i) there was any change in serum creatinine >26 μmol/L (0.3 mg/dL) from baseline or >50% from baseline, or (ii) there was any change in serum creatinine >26 μmol/L (0.3 mg/dL) with repeated measures within 24 hours. If confirmed, the participant was withdrawn, and further investigations were conducted.

### Liver chemistry stopping criteria

The Sponsor hepatic safety panel liver chemistry stopping and increased monitoring criteria were designed to assure participant safety and evaluate liver event etiology. Discontinuation of study treatment following abnormal liver tests was required when:

- A participant met one of the conditions outlined in the algorithm (see **S1** **Fig**) or,
- In the presence of abnormal liver chemistries that did not meet protocol-specified stopping rules, if the PI believed study treatment discontinuation was in the participant’s best interest.

**S1 Fig. Part I liver chemistry stopping criteria – liver stopping event algorithm**


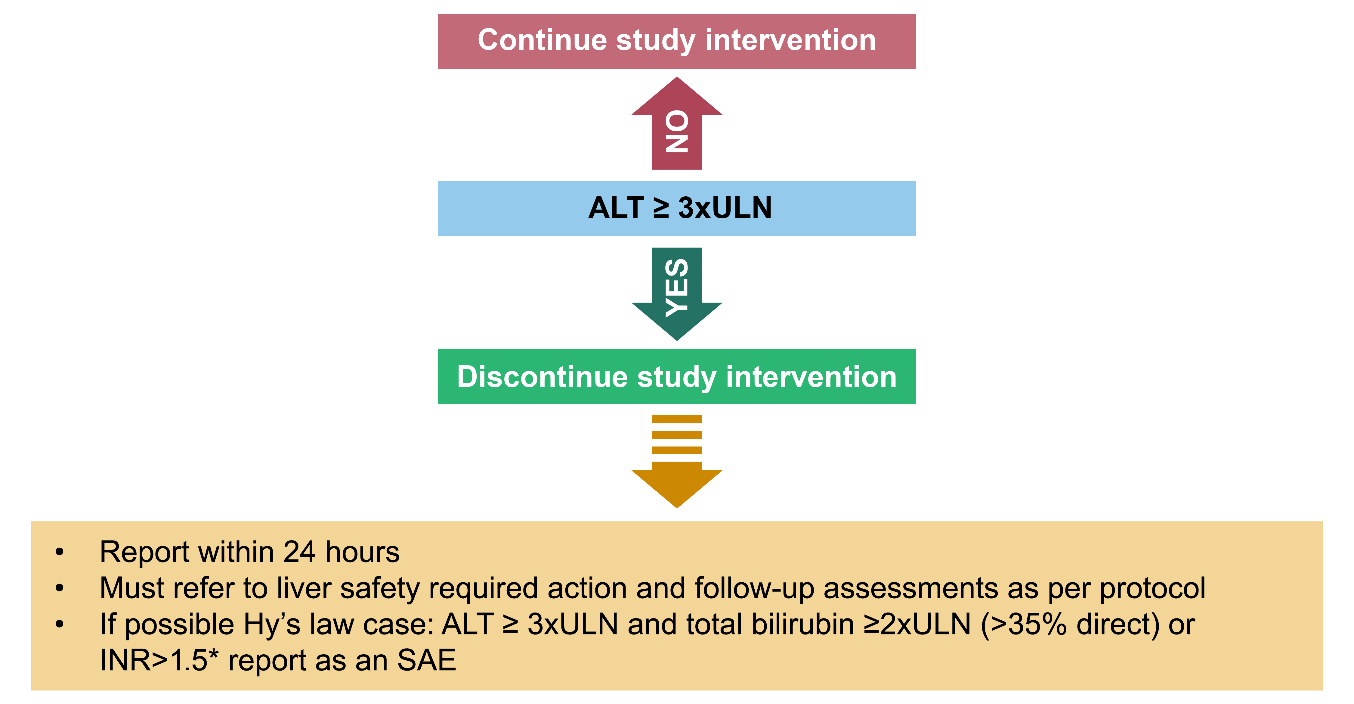


**Abbreviations:** ALT=alanine aminotransferase; INR=international normalized ratio; ULN=upper limit of normal; SAE=serious adverse event.

**Note:** *INR value did not apply to participants on anticoagulants.

### ECG and telemetry stopping criteria

A participant who met one of the criteria below, based on the average of triplicate electrocardiogram (ECG) readings, was withdrawn from the study treatment.

- Corrected QT interval (QTc) >500 msec,
- Change from baseline: QTc >60 msec, or
- Non-sustained ventricular tachycardia (NSVT) >3 beats or premature ventricular contraction (PVC) triplets on telemetry.

### Olfactory stopping criteria

The olfactory stopping criteria were defined as follows:

- New onset changes in smell that could not be explained by other causes, following further investigations, at the PI’s discretion and in consultation with the Sponsor Medical Monitor.

### Mitochondrial toxicity stopping criteria

The mitochondrial toxicity stopping criteria were defined as follows:

- Unexplained lactic acidosis as determined by the PI, in consultation with the Sponsor Medical Monitor.
- Clinical symptoms, signs, clinical chemistry, and hematology parameters were reviewed by the PI, Sponsor Safety and Medical Governance lead, and Sponsor Medical Monitor during dose escalation meetings. Following this review, a decision could be made to withdraw individual participants or stop the study based on potential mitochondrial toxicity.
  - If a participant developed acidosis (where the PI confirmed low pH) during the study, the following actions were considered: serum and urine ketones, chloride, and calcium measurements contemporaneously with low pH.
  - If an AE of acidosis was observed on a venous blood gas test, an arterial blood gas test was performed if deemed appropriate by the PI to inform the clinical care of the participant.

### Individual safety stopping and withdrawal criteria

The individual safety stopping and withdrawal criteria were defined as follows:

- If a participant experienced a serious or severe clinically significant AE that, in the PI’s clinical judgment, was possibly, probably, or definitely related to the study product.
- If a participant started treatment with any prohibited medications.
- If any renal or liver laboratory results, ECG, or telemetry, olfactory, or mitochondrial toxicity stopping criteria were met.
- If a participant developed stomach erosion as confirmed clinically and following a gastroenterology specialist’s opinion and endoscopy (if required).
- If a participant developed COVID-19-like symptoms during the study, the following actions were taken:
  - Participants who developed a high clinical index of suspicion for COVID-19 disease were isolated and tested for COVID-19 in accordance with the site procedures.
  - Assessments were continued as per the protocol during this period; the withdrawal of participants from the study was at the discretion of the principal PI, with prior discussion and agreement with the Sponsor Medical Monitor.

### Safety stopping criteria

In addition to the criteria specified above, AEs, SAEs, laboratory abnormalities, ECGs, and changes in vital signs occurring across all randomized participants were regularly reviewed by the Dose Escalation Committee to ensure appropriate participant safety. Any changes to the study due to safety reasons were promptly communicated to the appropriate regulatory authorities and an independent research ethics committee.

**Study Treatment Restart or Rechallenge**

If any stopping criteria were met by a participant in this study, the study treatment was not allowed to be restarted or rechallenged.
