# Supplementary material for: Safety and pharmacokinetics of GSK3494245, a highly selective Leishmaniasis kinetoplastid proteasome inhibitor for the treatment of visceral leishmaniasis: A Phase 1, randomized, single ascending dose escalation study in healthy participants
Source: PLoS Negl Trop Dis. 2026 May 13;20(5):e0014181. doi: 10.1371/journal.pntd.0014181 (PMC13245864; doi:10.1371/journal.pntd.0014181)
Supplement: S2 Text — (DOCX) [file pntd.0014181.s002.docx]

## S2 Text. Inclusion and exclusion criteria

### Inclusion criteria

Participants were eligible to be included in the study only if all of the following criteria applied:

1. Participant must be 18 to ≤55 years of age at the time of signing the informed consent form.

2. Healthy as determined by the PI or medically qualified designee based on a medical evaluation including medical history, physical examination, laboratory tests, and cardiac monitoring. A participant with a clinical abnormality or laboratory parameter(s) that was/were not specifically listed in the inclusion or exclusion criteria, outside of the normal reference range for the population being studied, were included only if the PI, in consultation with the Sponsor Medical Monitor (if required), agreed and documented that the finding was unlikely to introduce additional risk factors and did not interfere with the study procedures. Note: Participants with out-of-range laboratory values at screening could undergo a single repeat assessment for eligibility, at the discretion of the PI.

3. Body weight ≥50 kg and body mass index (BMI) within the range of 18.5–28 kg/m^2^ (inclusive).

4. Male participants only.

a. A male participant with a female partner of reproductive potential must agree to use contraception as detailed in the clinical study protocol during the intervention period and for at least 90 days after the last dose of study treatment and refrain from donating sperm during this period.

5. Capable of giving signed informed consent as described in the protocol, which includes compliance with the requirements and restrictions listed in the informed consent form and protocol.

### Exclusion criteria

Participants were excluded from the study if any of the following criteria applied:

1. History or presence of cardiovascular, respiratory, hepatic, renal, gastrointestinal, endocrine, hematologic, or neurological disorders capable of significantly altering the absorption, metabolism, or elimination of drugs; constituting a risk when taking the study treatment; or interfering with the interpretation of data.

2. Abnormal blood pressure, as determined by the PI.

3. Previous history of leishmaniasis.

4. Alanine transaminase (ALT) > upper limit of normal (ULN) at screening or day -1.

5. Total bilirubin >1.5×ULN (isolated bilirubin >1.5×ULN was acceptable if total bilirubin was fractionated and direct bilirubin <35%).

6. Current or chronic history of liver disease or known hepatic or biliary abnormalities (with the exception of Gilbert’s syndrome or asymptomatic gallstones).

7. Current or history of clinically significant gastritis or gastroduodenal ulcers or regular use of non-steroidal anti-inflammatory drugs (NSAIDs).

8. Consumption of >14 units/week of alcohol.

9. Current or history of change in taste or smell without any plausible clinical explanation based on the PI’s clinical judgment.

10. QTc >450 msec based on the average of triplicate ECGs.

11. Waveform abnormalities, including premature ventricular complex (PVC) triplets and more than 500 single PVCs in 24 hours, or any other abnormalities, at the discretion of the PI.

12. Medical history of cardiac arrhythmia or cardiac disease, or a family or personal history of long QT syndrome.

13. Past or intended use of over-the-counter or prescription medication, including herbal medications, NSAIDs, proton pump inhibitors (PPIs), or anti-H2 antagonists within 7 days (or 14 days if the drug is a potential enzyme inducer) or five half-lives (whichever is the longest) prior to dosing. Other concomitant medications were considered on a case-by-case basis by the PI in consultation with the Sponsor Medical Monitor. Paracetamol was permitted (capped to ≤2 g/day).

14. Participation in the study resulted in loss of blood or blood products of more than 500 mL within a 56-day period.

15. Exposure to more than four new chemical entities within 12 months prior to the first dosing day.

16. Current enrollment or past participation within the past 30 days before signing of consent to any other clinical study involving an investigational study intervention or any other type of medical research.

17. Current enrollment or past participation in this clinical study.

18. Participants with an age-appropriate eGFR <90 mL/min/1.73 m², as calculated using the Chronic Kidney Disease Epidemiology Collaboration (CKD-EPI) 2009 equation, were considered to have reduced renal function.

19. Screening urine albumin: creatinine ratio >30 mg/g (>3 mg/mmol).

20. Positive hepatitis B surface antigen (HBsAg) test result at screening.

21. Positive hepatitis C antibody test result at screening. Note: Participants with positive hepatitis C antibody due to prior resolved disease were enrolled, but only if a confirmatory negative hepatitis C ribonucleic acid (RNA) test result was obtained.

22. Positive hepatitis C RNA test result at screening. Note: Testing was optional, and participants with a negative hepatitis C antibody test were not required to undergo hepatitis C RNA testing.

23. Positive human immunodeficiency virus (HIV) antibody test.

24. Presence of clinically significant hematuria and/or proteinuria.

25. Carbon monoxide levels indicative of smoking, or history or regular use of tobacco or nicotine-containing products within 3 months prior to screening.

26. Positive pre-study drug/alcohol screen.

27. Regular use of known drugs of abuse.

28. Fed regimens only: Participant must have no dietary restrictions (e.g., lactose intolerance) or inability to eat an adapted standard meal (includes 35-40% fat content).

29. Fed regimen only: History of gall bladder surgery or gall bladder removal, or history of an acute disease state (e.g., cholelithiasis) within 14 days prior to receiving the study treatment.

30. Participants must not have traveled to an area (as determined by the PI) with a high prevalence of leishmanial/parasitic infections in the 6 months before screening or intend to do so in the 3 months after the final dose of study treatment.

31. Sensitivity to any of the study treatments, or components thereof, or drug or other allergy that, in the opinion of the PI or Sponsor Medical Monitor, contraindicates participation in the study.

32. A positive laboratory confirmation of COVID-19 infection [validated polymerase chain reaction (PCR) or lateral flow test], or high clinical index of suspicion for COVID-19.
